# A cohort study of gestational diabetes mellitus and complimentary qualitative research: background, aims and design

**DOI:** 10.1186/s12884-014-0378-y

**Published:** 2014-11-25

**Authors:** Vijayam Balaji, Madhuri S Balaji, Manjula Datta, Rekha Rajendran, Karoline Kragelund Nielsen, Rohini Radhakrishnan, Anil Kapur, Veerasamy Seshiah

**Affiliations:** Dr. V. Balaji Diabetes Care Centre - Dr. V. Seshiah Diabetes Research Institutes, # 729, P. H. Road, Aminjikarai, Chennai, 600 029 Tamil Nadu India; Madras Diabetes Research Foundation, Chennai, India; Department of International Health, Immunology and Microbiology, University of Copenhagen, Copenhagen, Denmark; World Diabetes Foundation, Gentofte, Denmark

## Abstract

**Background:**

Women with gestational diabetes mellitus (GDM) and their offsprings are at increased risk of future type 2 diabetes and metabolic abnormalities. Early diagnosis and proper management of GDM, as well as, postpartum follow-up and preventive care is expected to reduce this risk. However, no large scale prospective studies have been done particularly from the developing world on this aspect. The objective of this study is to identify and follow a cohort of pregnant women with and without GDM and their offspring to identify determinants and risk factors for GDM, for various pregnancy outcomes, as well as, for the development of future diabetes and metabolic abnormalities.

**Methods:**

This is a prospective cohort study involving pregnant women attending prenatal clinics from urban, semi-urban and rural areas in the greater Chennai region in South India. Around 9850 pregnant women will be screened for GDM. Socio-economic status, demographic data, obstetric history, delivery and birth outcomes, perinatal and postnatal complications, neonatal morbidity, maternal postpartum and offsprings follow-up data will be collected. Those diagnosed with GDM will initially be advised routine care. Those unable to reach glycaemic control with diet alone will be advised to take insulin. Postpartum screening for glucose abnormalities will be performed at months 3 and 6 and then every year for 10 years. The offsprings will be followed up every year for anthropometric measurements and growth velocity, as well as, plasma glucose, insulin and lipid profile. In addition, qualitative research will be carried out to identify barriers and facilitators for early GDM screening, treatment compliance and postpartum follow-up and testing, as well as, for continued adherence to lifestyle modifications.

**Discussion:**

The study will demonstrate whether measures to improve diagnosis and care of GDM mothers followed by preventive postpartum care are possible in the routine care setting. It will also map out the barriers and facilitators for such initiatives and provide new evidence on the determinants and risk factors for both GDM development and occurrence of adverse pregnancy outcomes and development of future diabetes and metabolic abnormalities in the GDM mother and her offspring.

**Electronic supplementary material:**

The online version of this article (doi:10.1186/s12884-014-0378-y) contains supplementary material, which is available to authorized users.

## Background

Prevalence of diabetes is increasing globally, particularly in the developing world with China and India contributing a major part of the increasing burden. A serious concern is that India is projected to have the highest population of people with diabetes in the world, by 2030 [1]. The rise in prevalence is attributed to aging population, urbanization, rising obesity, unhealthy diets and physical inactivity, in addition to the genetic predisposition of South Asians to diabetes [2]. While all these factors do contribute to the epidemic of diabetes, early life programming seems to play a facilitator role and prepare the ground for adult life risk factors to come into play. The ‘Foetal Origin of Disease’ hypothesis proposes that susceptibility to adult diseases may be influenced by gestational programming [3], whereby stimuli or stresses encountered by the foetus at critical or sensitive periods of development can permanently induce structural, physiological, and metabolic changes, which predispose the individual to disease in adult life [4].

Primary prevention of type 2 diabetes mellitus (T2DM) encompasses maintaining normoglycaemia in genetically or otherwise susceptible individuals [5]. Primary prevention strategies like lifestyle modification with or without pharmacological interventions can delay or prevent the development of T2DM in persons diagnosed with impaired glucose tolerance (IGT) [6,7]. An ideal target for such preventive interventions are women with history of gestational diabetes mellitus (GDM) and their children, as they are at a very high risk of developing diabetes, predominantly T2DM. GDM is defined as, ‘any degree of glucose intolerance with onset or first recognition during pregnancy’ [8,9]. A recent meta-analysis shows that women with GDM have an increased risk of developing T2DM (RR 7 43, 95% CI 4 79–11 51). Within 5 years of an index pregnancy complicated by GDM, women had a relative risk of 4.69, which more than doubled to 9.34 in those examined more than 5 years postpartum [10]. A study from India found that women with GDM had a 3-fold increased lifetime risk of developing T2DM compared to pregnant women without GDM 16 years after index pregnancy [11]. In an Indian population it has been shown that by 17 years of age, one-third of children born to GDM mothers have evidence of impaired glucose tolerance (IGT) or T2DM [12].

One third (33%) of women with GDM in India give a history of maternal diabetes [3]. *In-utero* exposure to hyperglycaemia has been shown to be associated with increased occurrence of IGT and defective insulin secretory response in later stages of life, independent of genetic predisposition to T2DM [13]. In addition, children exposed to maternal diabetes *in-utero*, are known to have higher risk of obesity and diabetes compared to their unexposed siblings, suggesting non-genetic factors for the increased risk amongst exposed offsprings [13,14]. This compelling evidence centralizes the role of the intra-uterine environment in inordinately increasing the risk of future T2DM and other metabolic abnormalities, and offers unique opportunities for primary prevention.

As the age of onset of diabetes is declining and the child bearing age increasing, it is not uncommon for women to have previously undiagnosed diabetes when they become pregnant; it is therefore important that all pregnant women be tested both early in the pregnancy to rule out overt diabetes, as well as later on in the 2nd and 3rd trimesters to detect GDM. Since, women with GDM are at a high risk of developing diabetes 5–10 years postpartum, follow-up of women with GDM after the delivery is crucial. This is emphasized by the fact that T2DM can be delayed or prevented in women with GDM by lifestyle modifications or modest-intermittent drug therapy [7]. Apart from its relevance in preventing intergenerational transmission of diabetes, GDM - being one of the most common medical conditions associated with pregnancy – is also highly relevant for the prevention of adverse pregnancy outcomes. Thus identifying women with GDM, and implementing interventional strategies aimed at controlling glycaemic status has implications for maternal and neonatal morbidity and mortality through reductions in abortions, stillbirths, obstructed labour, macrosomia, shoulder dystocia and pregnancy-induced hypertensive disorders, pre-eclampsia, postpartum haemorrhage, neonatal hypoglycaemia, jaundice, infant respiratory distress syndrome etc.

This article describes the study design and research methodology for the identification and follow-up of pregnant women with GDM and their offspring. The aim is to establish a cohort consisting of pregnant women in the state of Tamil Nadu to study and understand 1) determinants, e.g. socio-economic status, and risk factors for the development of GDM, 2) determinants for achievement of glycaemic control among women diagnosed with GDM, 3) differences in pregnancy-related outcomes among women with and without GDM and their offsprings, 4) determinants for various postpartum health indicators among women with GDM and their offspring, and 5) differences in postpartum health indicators among women with and without GDM and their offsprings. In addition to the cohort study, complimentary qualitative research will be carried out. This comprehensive study will help shape the public health response to the rising burden of GDM and diabetes.

## Methods

### Design

This prospective cohort study is designed to identify women with GDM, and follow them through pregnancy and for several years postpartum. A sample size of 1200 pregnant women with GDM is planned. With 13.4% reported prevalence of GDM [15], screening a cohort of at least 9850 pregnant women is expected to give approximately 1320 women with GDM. Anticipating a maximum dropout of 10%, about 1200 women with GDM will be available for a follow-up of 10 years. Measures such as reminder phone calls and house visits will be employed to ensure a high follow-up rate.

### Study setting

Screening for GDM during pregnancy has gradually over the last couple of years become a routine part of antenatal care in Tamil Nadu. The study will be carried out at three different health centres: Dr. V. Seshiah Diabetes Care and Research Institute, the Corporation Maternity Hospital and the Periyapalayam Government Primary Health Centre. The first centre is a private diabetes hospital located in urban Chennai, which caters mainly to the middle class population. The other two centres are government run and cater to the lower income groups in a semi-urban and rural setting, respectively. The sites have previously been used for GDM research [5]. The government of Tamil Nadu offers free antenatal care services through the government run health centres and almost all pregnant women in the state have at least three antenatal care visits and most have an antenatal care visit in the first trimester. Middle class pregnant women will usually attend private practicing gyn/obs for antenatal care, who will then refer the women for a routine GDM test at Dr. V. Seshiah Diabetes Care and Research Institute.

### Subject recruitment

All pregnant women attending one of the three centres will be enrolled in the study when attending GDM screening at the prenatal clinic at the government health centres or at Dr. V. Seshiah Diabetes Care and Research Institute, and undergo preliminary clinical examination. The recruitment will be conducted by trained field staff. Prior to the examination, subjects will be briefed about the purpose of the study, methods (vide infra) to be adopted and written signed informed consent (Additional file 1) will be obtained from each participant.

All pregnant women will be enrolled in this study, irrespective of their gestational age, parity, age, or socio-economic status. All women will undergo a test for GDM, irrespective of whether they have been tested for GDM previously or not, and the group will be divided in to those diagnosed with GDM and those with normal glucose tolerance. Women with known pre-gestational diabetes will be excluded. A GDM registry will be established and study data as described below will be collected from all subjects.

### Main exposure and outcome variables

As mentioned, a number of studies are planned based on this cohort. The key outcome variables will be diagnosis of GDM, various adverse pregnancy outcomes such as macrosomia, stillbirth, congenital abnormality, pre-eclampsia and postpartum metabolic abnormalities such as hyperglycaemia, hyperlipidaemia and anthropometric details of both the mother and child. The exposure variables will be various demographic and socio-economic details, obstetric history including previous pregnancy complications, anthropometric measurements and clinical parameters e.g. plasma glucose and lipid profiles in the various trimesters, treatment plan and postpartum breastfeeding.

### Data collection at recruitment and subsequent follow-up visits during pregnancy

The data collection will be performed by a team of field staff who will be trained on methods for data collection, anthropometric measurements, sampling of blood specimen, lifestyle and diet counselling, at the centre before the commencement of field work. This is to ensure that each team member understands and implements the procedure that he/she is expected to perform thereby reducing the intra- and inter-observer variability.

A proforma for data collection, including patients’ identification details, socio-economic status, demographic characteristics, obstetric history, delivery and birth outcomes, perinatal and postnatal complications, neonatal morbidity, maternal postpartum follow-up and the follow-up data of offsprings, has been developed (Additional file 2). The reliability of the proforma was tested in an unpublished pilot study where the proforma was administered on the same 25 pregnant women by two investigators independently. The inter-observer agreement of the proforma was very high (Kappa = 0.85; A Kappa value of >0.8 suggests good agreement between the observers).

**Figure 1:**
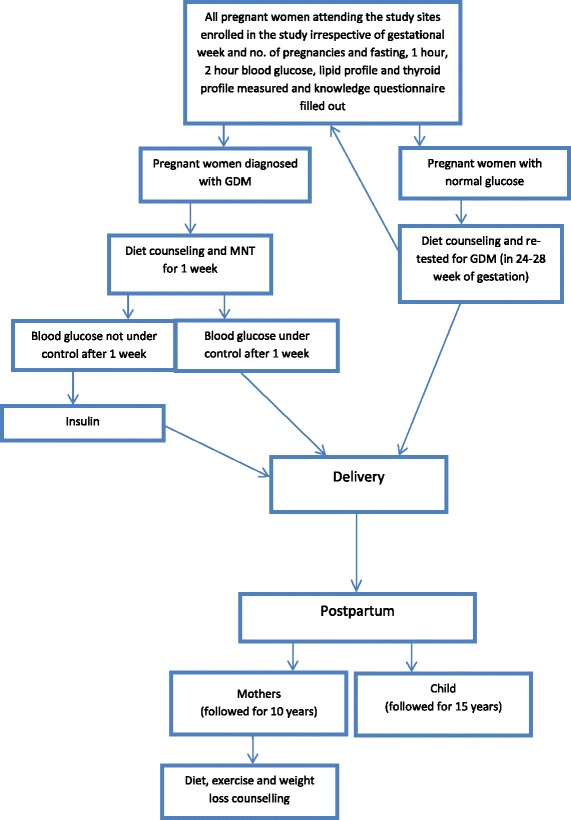
**Flowchart of the study.**

At the time of recruitment/first visit, the pregnant women’s identification and contact details, socio-economic status, demographic characteristics and obstetric history will be recorded. In addition, all study participants will undertake a knowledge and attitudes questionnaire (Additional file 3), which will guide health education, and laboratory and anthropometric measurements will be recorded. For women recruited in their first and second trimester of pregnancy, some of these measurements will be repeated when they return for a follow-up visit in the following trimester(s). The study design i.e. the participant flow and data collection is depicted in Figure 1 and Table 1.

**Table 1:** **Timing of collection of data**

**Time of data collection**	**Data collected**
Enrolment	− General information and identification details.
	− Socio-economic and demographic details.
	− Obstetric history.
	− Fasting & 2 h plasma glucose, 1 h blood glucose, HbA1c, HB, Thyroid profile, Lipid profile.
	− Height, weight, blood pressure.
	− Knowledge and attitudes questionnaire.
	− Foetal monitoring when possible.
Delivery	− Mode of delivery, birth weight, respiratory rate, ponderal index, APGAR score.
	− Hypoglycaemia, shoulder dystocia, respiratory distress.
	− Anthropometric details.
	− Cord blood to assess insulin, c-peptide and glucose.
3 months postpartum	− Breastfeeding practices.
	− Fasting and 2 h plasma glucose for the mothers.
	− Weight of the mothers.
	− Assessment of depression in the mothers.
6 months postpartum	− Breastfeeding practices.
	− Fasting and 2 h plasma glucose for the mothers.
	− Weight of the mothers.
	− Assessment of depression in the mothers.
1 year postpartum	− Breastfeeding practices.
	− Fasting and 2 h plasma glucose for the mothers.
	− Lipid profile for the mothers.
	− Weight of the mothers.
	− Assessment of depression in the mothers.
	− Weight, height and flank skin fold thickness of the children.
	− Fasting and 2 h plasma glucose for the children.
	− Insulin and lipid profile for the children.
2 years postpartum	− Breastfeeding practices.
Every year till year 10	− Fasting and 2 h plasma glucose for the mothers.
	− Lipid profile for the mothers.
	− Weight of the mothers.
Every 2nd year till year 15	− Weight, height and flank skin-fold thickness of the children.
	− Fasting and 2 h plasma glucose for the children.
	− Insulin and lipid profile for the children.

#### Laboratory measurements

After an overnight fast blood will be collected for the estimation of fasting plasma glucose (PG), following which all pregnant women will be administered 75 g anhydrous oral glucose dissolved in 150 ml of water and venous blood samples collected after 2 h for measuring PG levels. In the urban centre, blood samples will also be collected after 1 h of oral glucose administration. In the semi-urban and rural centres, first hour PG will be estimated using a calibrated glucometer [One touch® select®], as pregnant women in these areas may object to blood collection multiple times. Pregnant women will be considered to have GDM if 2-h PG ≥140 mg/dL (≥7.8 mmol/L), in accordance with the national policy and the Diabetes in Pregnancy Study Group in India (DIPSI) recommendation [16]. Pregnant women with normal glucose tolerance test in the first visit would undergo a repeat glucose challenge test around 24 to 28 weeks of gestation. Plasma glucose will be estimated by enzymatic glucose oxidase-peroxidase method in the central laboratory of Dr. V. Seshiah Diabetes Care and Research Institute using Hitachi fully automated analyzer.

A number of biochemical parameters will also be measured (see Table 2) from the fasting samples collected at the same visit(s) when the glucose tolerance test is performed. Lipid profiles and glycated haemoglobin will be estimated in all pregnant women during the first and third trimesters. Total cholesterol, triglycerides, low density lipoprotein-cholesterol, high density lipoprotein-cholesterol, C-reactive protein and fasting and post prandial insulin will be estimated in all pregnant women. Thyroid stimulating hormone levels will also be estimated.

**Table 2:** **Estimation of lipid profile, HbA1c, insulin, c-reactive protein and thyroid stimulating hormone**

**Parameter**	**Method of estimation**	**Equipment**
Total cholesterol	CHOD-PAP method	COBAS C311 & c501
Triglycerides	GPO-PAP method	COBAS C311 & c501
Low density lipoprotein-cholesterol	Friedewald *formula:*	NA
	LDL Cholesterol = (Total Cholesterol) – (HDL- Cholesterol) – (Triglyceride/5)	
High density lipoprotein-cholesterol	Phosphotungstic acid precipitation and enzymatic colorimetric method	COBAS C311 & c501
C-reactive protein	Sandwich principle Technique.	COBAS C311 & c501
Fasting insulin	CLIA technique	COBAS C311 & c501
Post prandial insulin	CLIA technique	COBAS C311 & c501
Thyroid stimulating hormone	Electro Chemilumiscent technique	COBAS C311 & c501
Glycated haemoglobin (HbA1c)	HPLC method	Variant II Turbo-2-BIORAD

#### Anthropometric measurements

Anthropometric measurements will be recorded for all pregnant women as described in Chennai urban population study [17]. At the urban centre these will be recorded at the time of laboratory measurements, whereas in the semi-urban and rural centres these will be done at the antenatal care visit preceding the laboratory measurements.

Height will be recorded in centimetres with the subjects standing against the wall in an upright position without footwear using a calibrated stadiometer. Weight will be measured in kilograms on a calibrated, electronic, digital weighing scale. Subjects will be requested to wear light clothing and stand upright without footwear.

Sitting blood pressure (BP) will be recorded from the right arm using a sphygmomanometer in a sitting position. The mean of two BP readings, taken 5 minutes apart will be recorded. Pregnant women will be categorized as hypertensive if they were/are on antihypertensive treatment or if they have a systolic blood pressure >140 mm Hg and/or diastolic blood pressure >90 mm Hg. Diagnosis of hypertension will be made according to the Joint National Committee (on prevention, detection, evaluation, and treatment of high blood pressure) criteria [18].

### Prenatal management

#### Nutritional and lifestyle modification

The nutritional and lifestyle recommendations used in this study follow the DIPSI guideline [16] and are similar to those routinely given in the participating health centres. A diet plan for each woman diagnosed with GDM will be distributed and explained by the dietician or trained field staff (Additional files 4 and 5). The diet plan among other things dictates no or reduced consumption of sugar and rice and high intake of greens and vegetables and adequate nourishment to maintain recommended weight appropriate for the gestational week. The counselling will take into consideration responses to the knowledge and attitudes questionnaire to ensure that the necessary information is provided and misconceptions are clarified. Care will be taken to ensure that pregnant women with GDM adhere to the diet plan throughout their pregnancy by repeating diet counselling at each visit. In addition, all women will be recommended 30 minutes of brisk walking every day [19].

#### Insulin therapy

Pregnant women with GDM who fail to reach the target 2-h PG <120 mg/dL with nutrition therapy within 2 weeks will be advised to take an appropriate dose of insulin in addition to the meal plan. The assessment will be carried out by the physician at the health centre and adjustments in the insulin dose will be made throughout pregnancy if necessary to achieve glycaemic control. Insulin therapy will by default be continued till delivery.

#### Foetal monitoring

If foetal ultrasound scanning has already been done during antenatal care visit, the information regarding estimated foetal weight, heart rate and amniotic fluid volume during the first trimester and at 18–20 weeks of gestation will be collected and recorded. The cervical length, weight gain and foetal abnormalities will be sought and recorded. Medical records from the antenatal care providers will be used to assess abdominal circumference.

### Intrapartum and postpartum management and data collection

Complications pertaining to the mother during pregnancy and delivery, e.g. pre-eclampsia and pregnancy induced hypertension will be assessed and recorded. Other adverse pregnancy outcomes such as abortion or stillbirth will likewise be recorded.

#### Intrapartum management

The intrapartum management of pregnant women will be undertaken as per the protocol of the local hospital in conjunction with physician, obstetrician and neonatologist, with main focus on glycaemic control and optimum blood pressure of the mother in case of GDM. Neonates will be monitored closely by the attending doctor who will note down information related to mode of delivery, birth weight, ponderal index, Appearance Pulse Grimace Activity Respiration (APGAR) score, shoulder dystocia, respiratory distress and hypoglycaemia. This information will be collected shortly after delivery by the field staff along with anthropometry details of the infant. In addition, cord blood will be collected for assessment of insulin, c-peptide and glucose levels. Early breastfeeding of neonates will be encouraged for all mothers. Since neonates of mothers with GDM are at risk of complications like respiratory distress syndrome, hypoglycaemia, hypocalcaemia and hyperbilirubinemia, they will be monitored closely for 24 h after birth. Macrosomia in the study will be defined as birth weight above 3.5 kg.

#### Postpartum management

All pregnant women – both those with GDM and those without - will be screened for diabetes and have their PG values assessed postpartum. Appropriate medical care of mother and child will be taken through education and intervention strategies.

#### Postpartum screening for diabetes

Glucose tolerance test will be performed at months 3 and 6, and thereafter once every year for 10 years postpartum as per the standard guidelines of WHO expert committee [20]. At the first postpartum visit women willing to participate in the long-term follow-up will be required to again sign a consent form, and will be handed a health card and information sheet with the contact details of investigators. In addition, lipid profile will be estimated at one year postpartum and every year thereafter, in order to ascertain the factors associated with future development of IGT, obesity, diabetes and cardiovascular diseases.

#### Lifestyle interventions

Mothers with GDM will be counselled regarding their higher risk of developing diabetes and necessity for postpartum follow-up. Women will be educated through pamphlets/hand-outs and one to one diet and lifestyle counselling for them and their children. They will be encouraged to continue implementing changes made during pregnancy in their diet and physical activity even after delivery.

In addition to diet counselling, the new mothers, both those with GDM and those without, will be encouraged for weight loss if required (BMI > 25). The new mothers will be encouraged to initiate early breastfeeding and continue it for ≥1 year. General diet advice for children of women with GDM will be provided.

#### Postpartum follow-up

The children of both, mothers with GDM and those without, will be followed-up for their weight, height, flank skin-fold thickness, OGTT, insulin and lipid profile at the age of 1 year and then every second year up to year 15. In case any markers of risk for diabetes, or cardiovascular disease, are identified, the mothers will be educated about preventive measures. If necessary, treatment will be initiated.

At 3 months, 6 months, and 1 year postpartum and then every year for 10 years, the weight of mothers will be measured. Also, at 3 months and 6 months postpartum mothers will be assessed for depression using a depression questionnaire adopted from Edinburgh postnatal depression scale (Additional file 6) [21,22]. Women falling in the depression score will be referred to a psychiatrist. The same depression assessment along with knowledge and attitude questionnaire will also be done one year postpartum. Mothers will be assessed for breastfeeding (Additional file 7) intensity at 3 months, 6 months, 1 year and 2 years postpartum. They will also receive counselling on family planning and birth control as per the local state policy. Subjects lost to follow-up will be contacted through phone, e-mail and text SMS or by home visits as a last resort and encouraged to continue follow-up.

### Planned statistical analysis and quality control

All data pertaining to the study population, starting from their first visit until the last follow-up will be documented and stored using the Epi Info software. Data will be analysed using statistical software, SPSS (version 16). The integrity of data collected will be reviewed weekly and missing and erroneous data will be corrected immediately. Descriptive analysis will include simple 2×2 tables, where data on continuous variables are presented as mean ± standard deviation (SD) and categorical variables are presented as number (%). Significance will be assessed at a 5% level. Chi-square/Fisher Exact test will be used to find the significance of between group differences in study parameters on categorical scale. In addition, Student *t*-test will be used to determine the significance between the means of two groups for various study parameters. When relevant, the odds ratio will be calculated. Multivariate regression analysis will be used to explore the relationship between various independent variables and the dependent variable; for instance, between various socio-economic factors, demographic parameters, obstetric history and various clinical parameters; and prenatal and postnatal hyperglycaemia.

### Complimentary qualitative research

Apart from the establishment of the cohort study, qualitative research will also be carried out. This research will focus on identifying barriers and facilitators for early GDM screening, treatment compliance and postpartum diabetes testing, and continued lifestyle modifications. Pregnant women who have or were supposed to have attended GDM screening will be interviewed and focus group discussions with such women will also be organized. In addition, women diagnosed with GDM will be interviewed during pregnancy and again postpartum. The women will be recruited through health care centres in urban and rural areas, and also through village health nurses in rural areas. Recruitment will be continued until theoretical saturation has been reached. Moreover, individual interviews with health care providers as well as observation at the centres will also be conducted. The qualitative data will be analysed using content analysis.

## Discussion

This cohort study includes pregnant women attending services for GDM testing at three clinics in greater Chennai, including an urban private hospital setting, a semi-urban government hospital setting and a rural government hospital setting. It was previously reported that the prevalence of GDM using the adapted WHO 1999 guideline as recommended by DIPSI in these areas ranged from around 10% to 18% [5]. This unique study will provide important data to investigate the role of various risk factors for GDM. It is also expected to shed light on the role of various biochemical, clinical and social factors on adverse pregnancy outcomes, including macrosomia. Finally, it will provide much needed details pertaining to the long-term outcomes in both women with GDM and their offsprings. To our knowledge, this is one of the first cohorts established to make such detailed quantitative and qualitative investigations on GDM in a developing country and the main strength of this study is its long duration. The need for such research has recently been highlighted by the WHO in their publication of the new guideline for diagnosing hyperglycaemia first detected in pregnancy [20].

Though we believe there is a great need for research like this from developing countries, we also acknowledge that there will be challenges in implementing such an ambitious cohort study. One hurdle is that there is no comprehensive and detailed surveillance system in place in the state, which may make it difficult to track the mothers and their offsprings, particularly in the Indian health system which is a complex intermix of private and government health care providers and patients floating in and out freely between them. The situation is further complicated by the fact that pregnant women tend to move to their native home for delivery [23] and therefore may not be visiting the same prenatal clinic in the beginning and in the end of their pregnancy. Moreover, some pregnant women may go to a private clinic to have ultrasonography, but attend a government clinic for general antenatal check-ups.

A number of studies have shown that rates of postpartum follow-up of women with GDM are often at an unsatisfactory level [24-29]. Studies from Canada and the US found that women with a history of GDM reported time pressure, lost requisition, dissatisfaction with health care services, logistics of accessing care, believing not to be in need of care, fear of receiving bad news, emotional stress, feeling overwhelmed, burden of child care, baby’s health issues and recent delivery experiences as barriers for attending postpartum diabetes testing [30,31]. No such studies have been conducted in India, but experiences from GDM projects supported by the World Diabetes Foundation, including projects in India, indicate the fact that a woman is considered lost in the system when she is no longer seen by either the obstetrician or the diabetes specialist. Women being too busy taking care of the child and doing other household work were reasons for not attending postpartum follow-up visits [23]. Acknowledging the challenge of ensuring a high postpartum follow-up rate, field staff have been trained to pay particular attention to this and to inform women of the importance of returning for follow-up. Reminders through phone, e-mail, SMS or house visits are planned. In addition, a close collaboration with the maternal and child health service providers in the areas has been established to further ensure a good postpartum follow-up rate through reminders during visit for the child’s vaccination.

In addition to the cohort study, qualitative research will be done to compliment the quantitative findings. The knowledge gained from this combined research will be used to further improve GDM related detection, counselling, treatment and postpartum follow-up. The findings from the study will be used for awareness and advocacy and further shape and enhance the public health response to the rising burden of GDM and T2DM in India and in other developing countries.

## Ethical approval

Ethical approval has been obtained from the Institutional Ethics Committee at Dr. Balaji Diabetes Care Centre and Dr. V. Seshiah Diabetes Research Institute. Since the complimentary qualitative research is carried out by a Danish researcher an inquiry was made to the Danish Committee System on Health Research Ethics which confirmed that the qualitative studies are exempt from ethical approval as they are interview-based studies without the use of human biological materials.

## Additional files

Additional file 1:
**Consent form.**
Additional file 2:
**Prevention of diabetes in mother and child project (PDMCP) - Study Proforma.**
Additional file 3:
**World Diabetic Foundation and Dr. V. Seshiah Diabetic Research Institutes & Dr. Balaji Diabetic Care Centre Prevention of Diabetes in Mother and Children Project.**
Additional file 4:
**Diet plan page 1.**
Additional file 5:
**Diet plan page 2.**
Additional file 6:
**Depression Assessment Questionnaire.**
Additional file 7:
**World Diabetic Foundation and Dr. V. Seshiah Diabetic Care & Research Institutes Prevention of Diabetes in Mother and Children Project - Brief Breastfeeding Questionnaire.**
