# Supplementary material for: A cohort study of gestational diabetes mellitus and complimentary qualitative research: background, aims and design
Source: BMC Pregnancy Childbirth. 2014 Nov 25;14:378. doi: 10.1186/s12884-014-0378-y (PMC4248438; doi:10.1186/s12884-014-0378-y)
Supplement: Additional file 1: — Consent form. [file 12884_2014_378_MOESM1_ESM.pdf]

Consent /Assent for Taking Informed Consent/Assent from Eligible Respondent for Participating in prevention of Diabetes in Mother and Children Project

Principle Investigator: Dr.V.Balaji

This form explains the purpose of the study, and why blood specimen is being collected and the method of blood collection. On reading / understanding the following information if you are willing to participate in the study and provide blood specimen you are request to sign or make a thumb impression at the end of form. If you have any questions queries you can ask us before giving the consent/assent.

World diabetes foundation (WDF), the international agency for control of Diabetes conducts research in various populations to know the prevalence of diabetes all over. The world Emergence of diabetes in pregnancy is focused in this study. Results from this will help WDF to develop appropriate program to control diabetes during pregnancy in your community, region and in India as a whole. 3000 people will be included in our study to represent our area and you are chosen to be one among them. If you agree to participate in the study few ml of blood will be collected before and 2 hour after breakfast. We will use disposable sterile instruments that are clean and completely safe.

We will ask you set of questions which are necessary for the study and all the information you provide will be confidential. Your honest answer to questions will help us better to understand the risk behind the disease.

Your decision to agree or refuse to participate will not affect the province of service under the targeted intervention project.

Do you have any questions?

I -----------------------------wife of----------------------------aged------ am willing to participate and provide blood samples for the study by my own wish. I know that this data will be utilized by WDF with full confidentiality.

Signature/thumb impression------------------------ Date---------------

This is the thumb impression of---------------------- Field investigator name----------------------

Name of witness--------------------- Signature--------------------------- Date---------------

Field investigator name------------- Signature--------------------------- Date--------------

தாய் மற்றும் குழந்தை நீரிழிவு நோய் தடுப்பு ஆராய்ச்சியில் பங்கேற்க தகுதியான பதிலளிப்பாளர்

தெரியப்படுத்தப்படும் அனுமதி / இசைந்து எடுத்து ஒப்புதல் / இசைந்து

கொள்கை சோதனையாளர்: Dr.வி.பாலாஜி

இந்த படிவம் ஆய்வு நோக்கத்தை, மற்றும் ஏன், எப்படி இரத்த மாதிரி சேகரிக்கப்படும் என்பதை விளக்குகிறது. நீங்கள் ஆய்வில் பங்கேற்க மற்றும் இரத்த மாதிரி வழங்க தயாராக இருந்தால் படித்து / பின்வரும் தகவல்களை புரிந்து கொள்ள நீங்கள் படிவத்தை இறுதியில் கையெழுத்து அல்லது விரல் ரேகை செய்ய கோரிக்கை உள்ளன. நீங்கள் ஏதேனும் கேள்வி கேட்க விரும்பினால் கையெழுத்திடும் முன் கேளுங்கள்.

சர்க்கரை நோய் கட்டுப்பாட்டில் சர்வதேச நிறுவனமான உலக நீரிழிவு அடித்தளம் (WDF), அனைத்து மேலாக நீரிழிவு நோய் பற்றிய ஆராய்ச்சிகளை பல்வேறு மக்கள் தொகையில் நடத்துகிறது. கர்ப்ப கால நீரிழிவு நோயின் உலக வெளிப்பாட்டை இந்த ஆய்வு மையமாக கொண்டுள்ளது.

இந்த முடிவுகள் உலக நீரிழிவு நிறுவனத்திற்கு, உங்கள் சமூகம், பகுதி மற்றும் முழு இந்தியாவின் கர்ப்ப காலத்தில் நிகழும் நீரிழிவு நோயை கட்டுப்படுத்துவதற்கு பொருத்தமான திட்டத்தை உருவாக்குவதற்கு உதவும். 3000 மக்கள் நம்முடைய பகுதியில் பிரதிநிதியாக தேர்ந்தெடுக்கப்பட்ட நிலையில் நீங்களும் ஒருவர். நீங்கள் ஆய்வில் பங்கேற்க புகுபதிவு ஒப்புக்கொண்டால் சில மில்லி இரத்தம் காலை உணவிற்கு முன்பு மற்றும், காலை உணவிற்கு 2 மணி நேரம் பிறகு எடுக்கப்படும். நாங்கள் நுண்ணுயிரற்ற முழுமையாக சுத்தமான பாதுகாப்பான செலவழிப்பு கருவியை மட்டுமே பயன்படுத்துவோம்.

நாங்கள் ஆய்வு செய்ய தேவையான கேள்விகளை கேட்டு, கிடைக்கும் தகவலை இரகசியமாக வைத்துக்கொள்வோம். உங்கள் நேர்மையான பதில் எங்களுக்கு சிறப்பாக நோயின் பின்னால் இடர்கள் புரிந்துகொள்ள உதவும்.

ஏற்றுக்கொள்ளப்பட்ட அல்லது மறுக்கப்பட்ட உங்கள் முடிவு ஏதுவாக இருப்பினும் அது சேவை மாகாணத்தில் குறிவைத்த தலையீடு திட்டத்தின் கீழ் ஆராய்ச்சியை எந்த விதத்திலும் பாதிக்காது.

உங்களுக்கு ஏதேனும் கேள்விகள் கேட்க வேண்டும்மா?

நான்-----------------------------மனைவியான----------------------------வயது--------- என் சொந்த விருப்பப்படி பங்கேற்க மற்றும் ஆய்வு ரத்த மாதிரிகள் வழங்கவும் புகுபதிவு செய்ய தயாராக இருக்கிறேன். இந்த தகவல்களை உலக நீரிழிவு அடித்தளம் (WDF), முழு இரகசியமாக பயன்படுத்தும் என்று எனக்கு தெரியும்.

கையொப்பம் / விரல் ரேகை --- தேதி ------------------------------

கையொப்பம் / விரல் ரேகை --- புல ஆராய்ச்சியாளர் பெயர்--------------------

சாட்சி பெயர்---------------- கையொப்பம் -- தேதி ------------------------------

புல ஆராய்ச்சியாளர் பெயர்-------------- கையொப்பம்---------- தேதி----------------
