# Supplementary material for: A cohort study of gestational diabetes mellitus and complimentary qualitative research: background, aims and design
Source: BMC Pregnancy Childbirth. 2014 Nov 25;14:378. doi: 10.1186/s12884-014-0378-y (PMC4248438; doi:10.1186/s12884-014-0378-y)
Supplement: Additional file 2: — Prevention of diabetes in mother and child project (PDMCP) - Study Proforma. [file 12884_2014_378_MOESM2_ESM.pdf]

ILD: ☐☐ / ☐☐☐☐

PREVENTION OF DIABETES IN MOTHER AND CHILD PROJECT (PDMCP) - STUDY PROFORMA

Date of Interview ___ /____/ ____
DD MM YY.

______________________________ **Health Centre**

**General Information**		
1	Name (in capital letters)	
2	Date of Birth	D D M M Y Y Y Y
3	Age (yrs)	
4	Occupation*	
5	Activity Level<br>1. Sedentary 2. Moderate<br>3. Heavy	
6	Educational status*	.
7	Commutation to health centre<br>Or work *	
8	Type of Family<br>1.Nuclear 2. Joint	
8.1	Family Income per month*	.
8.2	Number of years married	
8.3	Number of people in household	
9	Husband name	
10	Husband's Educational status*	
11	Husband's occupation*	
12	Complete address with landmark	
12.1	Contact No: Landline	
12.2	Contact No: Mobile	
12.3	Email ID	

Identification details		
13	Area name & code	
13.1	Area type<br>Urban-1, Semiurban-2, Rural-3	
14	Women serial number (MRD)<br>Centre specific	
15	Blood sample code (Lab No)	
16	Date of blood sample collection	D D M M Y Y
16.1	Field Investigator name & code	
16.2	Lab technician name & code	
17	Photocopy of Ration card / Voter's ID/ Adhar card	Yes ☐ No ☐
18	Name of the relative	
19	Relationship	
20	Address of relative	
20.1	Contact no: Landline	
20.2	Contact no: Mobile	
20.3	Email ID	

**Obstetric History**				
21	Gravida			
22	Parity			
23	Number of children alive today			
24	No of times Aborted			
25	Time since last pregnancy		Y	M
26	Gestational age at entry(Wks)			
27	Gestational age at diagnosis of GDM(Wks)			
28	Singleton or multiple pregnancy<br>Singleton-1, twins-2, more than 2-3			
29	Family history of Diabetes<br>Yes-1, No-2<br>If yes, mention<br>Father-1, Mother-2, Sibbling -3, Grandparents-4 1st degree-5, 2nd degree-6			
30	Previous known DM, IGT or FGT<br>No -1, if yes, DM -2, IGT-3, IFG-4			
31	Consanguinity Marriage<br>Yes-1, No-2<br>If yes, mention<br>1st degree-1, 2nd degree-2, 3rd degree-3			

No.	Item		HIV	Malaria	Hepatitis B	Hepatitis C	TB
32	Known history of infectious disease<br>Yes-1, No-2, Don’t know-3.	Yes					
		No					
		Don’t know					

33	Diagnosis of polycystic ovarian syndrome<br>Yes-1, No-2, don’t know-3		
34	Are you currently taking any medication Yes-1, No-2<br>If yes, please list medication and condition it is taken for		
35	Dietary habits<br>Veg -1, Non-veg -2		

ILD: ☐☐ / ☐☐☐☐

36. Details of Previous pregnancies

**Preg nancy**	**Outcome:** Live born -1 Still Born -2 Abortion -3 Dead within 1 yr -4	**Maternal Complications Yes-1, No-2**										
		**GDM**	**PIH**	**Pre-eclampsia**	**Eclampsia**	**Protein uria**	**Thyroid**	**Treatment**		**Other Information**	**Blood group A+, A-, B+, B-, AB+, AB-, O+, O-**	
								**Yes ***	**No**		**Mother**	**Child**
1												
2												
3												
4												
5												

***-** If yes, what was the treatment. --

37. Details of Previous deliveries (live born) and outcomes

**Pre gna ncy**	**Date of Delivery** (DD/MM/ YY)	**Place of delivery 1-health centre, 2-Home, 3-Maternal home, 4-Others**	**Gesta-tional age** (wks)	**Term** Preterm -1 Term-2	**Mode of Delivery** Normal-1 Induced-2 Planned LSCS-3 Emergency LSCS - 4	**Sex Of child**	**Birth Weight** (grams)	**Child Complications**			
								**Congenital Abnormalities** Yes -1, No - 2	**Other Complications*** Yes -1, No- 2 If yes, mention	**HIV** Yes-1 No -2	**Jaundice** Yes -1, No - 2

***-** If yes, what was the complication and at what age of the child---

LMP:	DD	MM	YY
EDD:			

**38. Maternal Details- Current Pregnancy**			
**38.1 Anthropometry & Clinical Parameters**	Pre pregnancy wt:	BMI:	
**Height of the Mother (cm):**			.

Trimester	GA (wks)	Date of Test	Wt (Kgs)	BMI	Body fat (%)	BP (mmHg)	
						SBP	DBP
**I**							
**II**							
**III**							

38.2 Plasma Glucose

Trimester	GA (wks)	Date of Test	Plasma Glucose (mg/ dl)				Hb%
			FPG	1 hr PG	2 hr PG	A1c	
**I**							
**II**							
**III**							

38.3 Lipid Profile

Trimester	GA (wks)	Date of Test	Lipid Profile						
			TC	TG	LDL	HDL	FFA	Glycerol	Lipoproteins
**I**									
**II**									
**III**									

38.4 Thyroid Profile

Trimester	GA (wks)	Date of Test	Thyroid Profile		
			T3	T4	TSH

38.5 Special Test at Entry Level

Trimester	GA (wks)	Date of Test	Adiponectin	c- RP	Insulin	Folic acid	Vit B12
**I**							
**II**							
**III**							

38.6 Ultra sonography Findings

Particulars					
Gestational week					
Fetal weight					
Fetal abdominal circumference					
Fetal heart rate					
Fetal length					
Fetal head Circumference					
Abdominal interpretations<br>Normal-1, Abnormal-2					

38.7 Treatment Plan

Trimester	GA (wks)	Diagnosis Yes -1, No-2				Treatment Yes -1, No -2					
		Pre-GDM	GDM*	GGI**	NGT***	MNT♣	OHA#		Insulin		Exercise
							Sul##	Met###	HI¶	Analogs	
I											
II											
III											

* Gestational Diabetes Mellitus
** Gestational Glucose Intolerance
*** Normal Glucose Tolerance (Less than 140 mg/dl for 2hour OGGT)
♣ Medical Nutrition Therapy
Oral Hypoglycemic Agent
¶ Human Insulin
Sulphonylurea
Metformin

Insulin Chart

Date	GA (wks)	Treatment	Dose	Duration

39. Current Pregnancy- Maternal complications			
39.1	Proteinuria	Yes -1, No-2, if yes, define which	
39.2	Pregnancy Induced Hypertension [PIH]	Yes -1, No-2, if yes, define which	
39.3	Pre- eclampsia	Yes -1, No-2, if yes, define which	
39.4	Retinopathy	Yes -1, No-2, if yes, define which	
39.5	Nephropathy	Yes -1, No-2, if yes, define which	
39.6	Post partum depression	Yes -1, No-2, if yes, define which	
39.7	Urinary tract infection	Yes -1, No-2, if yes, define which	
39.8	Others(specify)	Yes -1, No-2, if yes, define which	
39.9	Hospitalized before delivery due to complications	Yes -1, No-2, if yes, for which condition	
39.10	HIV	Yes -1, No-2, if yes, for which condition	

40. Outcome of Present Pregnancy			
40.1	Delivered live born baby Yes -1, No-2		
40.2	If No, how Was it wasted [Reason]<br>Still born -1<br>Abortion -2<br>Other -3 (specify),		
If Delivered,			
41	Date of Delivery	D D M M Y Y	
41.1	Gestational age (wks)		
42	Sex of the Child Male-1 Female-2		
43	Mode of Delivery<br>Spontaneous-1, Induction-2, planned LSCS-3, emergency LSCS-4		
43.1	Place of Delivery<br>Home-1, Institutional-2, Maternal home -3, Other-3 (specify)		
44	Birth Weight (gms)		
45	Height (cms)	.	
46	Head Circumference (cms)	.	
47	Chest Circumference (cms)	.	
48	Umbilical Circumference (cms)	.	
49	Percent Body fat		
50	Fat Free Mass (gms)		
51	Fat Mass (gms)		

52	Apgar Score (At 5 mins)	1min			5mins		
53	Ponderal Index						
54	New born infants blood glucose						
55	Cord blood insulin level						
56	Cord blood c peptide level						

57. Neonatal Body composition

Age during measurement (days) :

Extremities	Length (cms)	Circumference (cms)	Skin folds (mms)	
Arm			Triceps	
Forearm			Sub scapular	
Thigh			Flank	
Lower Leg			Thigh	

58. Neonatal Morbidity

Age during measurement(weeks):

58.1	Congenital Abnormality	Yes -1, No-2, if yes, define which	
58.2	Pre term	Yes -1, No-2	
58.3	Low Birth Weight	Yes -1, No-2	
58.4	Neonatal Hypoglycemia	Yes -1, No-2, if yes, define which	
58.5	Neonatal Jaundice	Yes -1, No-2, if yes, define which	
58.6	Shoulder Dystocia	Yes -1, No-2, if yes, define which	
58.7	Respiratory Distress	Yes -1, No-2, if yes, define which	
58.8	Hypocalcaemia	Yes -1, No-2, if yes, define which	
58.9	Hypovolemia	Yes -1, No-2, if yes, define which	
58.10	Polycythemia	Yes -1, No-2, if yes, define which	
58.11	Fetal growth group<br>SGA -1, LGA -2, AGA -3		
58.12	Admission for NICU	Yes -1, No-2	
58.13	Any other information	Yes-1 *, No-2.	

*-Yes – Add note --
----

59. Maternal Post- Partum Follow-up

Lactation stopped at (months):

Post partum depression: Yes -1, No-2, if yes,

Visit log	Date of visit	Lactation [Y/ N]	Wt [kg]	BMI	Body fat [%]	Plasma Glucose [mg/ dl]				Diagnosis [Normal/ IFG/ IGT/ Diabetes]	TSH	Lipid Profile							Adiponectin	Preventive treatment for DM*
						HB %	FPG	PPG (2 hr)	A1c			TC	TG	LDL	HDL	FFA	Glycerol	Lipo Protein		
3 months																				
6 months																×	×	×	×	
1 year																×	×	×	×	
2 year																×	×	×	×	
3 year		NA														×	×	×	×	
4 year		NA														×	×	×	×	
5 year		NA														×	×	×	×	
6 year		NA														×	×	×	×	
7 year		NA														×	×	×	×	
8 year		NA														×	×	×	×	
9 year		NA														×	×	×	×	
10 year		NA														×	×	×	×	

No treatment-0, MNT-1, MNT+Sul-2, MNT+Met-3, MNT+Gly-4, MNT+Combi-5, MNT+Insu-6

ILD: ☐☐ / ☐☐☐☐

60. Child Follow- up

Visit log	Date of Test	Anthropometric details					Plasma Glucose				Insulin	Lipid profile								Comments
		Ht	Wt	BMI	Body fat [%]	Fat mass [gm]	Hb %	Glucose Used for GTT	FPG	2 hr PPG	Fasting	TSH	TC	TG	LDL	HDL	FFA	Glycerol	Lipo protein	
1 year																				
3 year																				
5 year																				
7 year																				
9 year																				
11 year																				
13 year																				
15 year																				

Key

<table>
<tr><td>4. Occupation

1) Agricultural Labourer
2) Non_ Agricultural Labourer
3) Domestic Servant
4) Skilled / Semi Skilled Worker
5) Petty Business / Small Shop
6) Large Business / Self Employed
7) Service (Govt. / Pvt.)
8) Student
9) Truck Driver / Helper
10) Local Trasport Worker (Auto/Taxi Driver/Handcart Pullers/Rickshaw Pullers etc)
11) Hotel Staff
12) Agriculture Cultivator / Landholder
13) Unemployed
14) House Wife</td>
<td>7. Commutation to health centre? Or work?

1.Public transport
2.Two wheeler
3.threewheeler/Auto
4. Car
5. Walking
<hr>8.1 Family Income per month

1) No Income,
2) Upt o 5000
3) 5001 to 10000,
4) 10001 to 15000,
5) 15001 to 2000,
6) 20001 to 30000,
7) > 30000</td></tr>
<tr><td>5. Activity level

Sedentary: One with no brisk physical activity. E.g.: Watching TV, at rest often.

Moderate: Moderate level of physical activity with half an hour brisk walking, 10 minutes of exercise that breaks into sweat every day. E.g.: Mowing lawn, riding a bike on level surfaces and playing doubles tennis.

Heavy: Doing physical exercise vigorously for at least one hour and 15 minutes every week. E.g.: Biking or hiking on hills, jogging, swimming laps, playing basketball or high-intensity aerobics.</td>
<td>10. Educational status

1.Illiterate,
2. Literate and till 5th Standard
3. 6th to 10th Standard
4. 11th to 12th
5. 12th to Graduation,
6. Post Graduation</td></tr>
</table>

6. Educational status	11. Husbands occupation
1. Illiterate,<br>2. Literate and till 5th Standard<br>3. 6th to 10th Standard<br>4. 11th to 12th<br>5. 12th to Graduation,<br>6. Post Graduation	1) Agricultural Labourer<br>2) Non_ Agricultural Labourer<br>3) Domestic Servant<br>4) Skilled / Semi Skilled Worker<br>5) Petty Business / Small Shop<br>6) Large Business / Self Employed<br>7) Service (Govt. / Pvt.)<br>8) Student<br>9) Truck Driver / Helper<br>10) Local Trasport Worker (Auto/Taxi Driver/Handcart Pullers/Rickshaw Pullers etc)<br>11) Hotel Staff<br>12) Agriculture Cultivator / Landholder<br>13) Unemployed
