# Supplementary material for: A cohort study of gestational diabetes mellitus and complimentary qualitative research: background, aims and design
Source: BMC Pregnancy Childbirth. 2014 Nov 25;14:378. doi: 10.1186/s12884-014-0378-y (PMC4248438; doi:10.1186/s12884-014-0378-y)
Supplement: Additional file 3: — World Diabetic Foundation and Dr. V. Seshiah Diabetic Research Institutes & Dr. Balaji Diabetic Care Centre Prevention of Diabetes in Mother and Children Project. [file 12884_2014_378_MOESM3_ESM.pdf]

World Diabetic Foundation and Dr.V.Seshiah Diabetic Research Institutes & Dr Balaji Diabetic Care Centre Prevention of Diabetes in Mother and Children Project

Identification number	:	[ ][ ] [ ][ ][ ][ ]
Date of Interview	:	___ /____/ ____ (DD MM YY)
Name (In capital letters)	:	
Age (Yrs)	:	
Husband name	:	
Address	:	--
		--
		--
		--
Researcher Name	:	--
Centre of interview	:	Urban Semi urban Rural

1. What do you think of diabetes?
   a) Terrible/ dangerous disease
   b) Contagious
   c) Disease of the rich
   d) Ordinary disease
   e) Hereditary
   f) Don't know

2. Is there anyone in your family or any one you know have/has diabetes?
   a) Yes
   b) No
   C) Don't know

3. Whom do you think are more prone to diabetes?
   a) Men
   b) women
   C) Both
   d) Anybody
   e) Don't know

4. From whom do you think one can get Diabetes?
   a) Father
   b) mother
   c) from both
   d) on its own
   e) Husband/Wife
   f) Eating more sweet
   g) Don't know

5. What do you think are the symptoms of diabetes?
   a) Frequent urination
   b) Very hungry
   c) Non healing of wounds
   d) Itching in the private parts
   e) Loss of weight
   f) Very thirsty
   g) Tiredness
   h) Dimness in eye sight
   i) Don't know

6. What do you think happens if diabetes is not treated or controlled over long time or what organs are affected?
   a) Eye
   b) Nerves
   c) Limb removal
   d) Heart
   e) Kidney
   f) Don't know

7. During which stage of life do you think women are likely to get diabetes?
   a) During Menarche
   b) Menopause
   c) Any time
   d) During pregnancy
   e) Old age
   f) Don't know

8. How can you know whether a women has diabetes or not during pregnancy?
   a) Blood test
   b) Don't know
   c) Symptoms

9. What will be the mode of delivery and outcome of pregnancy for a woman with untreated or undiagnosed GDM?
   a) Normal delivery
   b) Caesarian
   c) Still birth
   d) Big baby
   e) Don't know
   f) Forceps delivery
   g) Premature delivery
   h) Neonatal death
   i) Handicapped child

10. If a woman is having uncontrolled/ undetected diabetes during pregnancy, is their or who is at risk of developing diabetes in future?
   a) Child will develop diabetes latter on life
   b) The mother will continue diabetic
   c) Both will get diabetes
   f) Mother will not become Diabetic
   g) The child will not become diabetic
   h) Both will not have diabetes

11. Do you think we can cure diabetes fully?
   a) We can
   b) We can't
   d) can only be controlled
   e) Don't know

12. What do you think one should do to keep diabetes under control?
   a) Diet control
   b) Exercise
   c) Do not need treatment
   f) Alternative medicines like herbs
   g) Approach a doctor
   h) Don't know

13. If the doctor suggests insulin injection, can we take it?
   a) Yes b) No C) Don't know

14. During pregnancy what were the test done for women?
   a) VDRL
   b) Hb%
   f) GDM
   g) HIV I & II
   h) Others specify----------------------------------
