# Supplementary material for: A cohort study of gestational diabetes mellitus and complimentary qualitative research: background, aims and design
Source: BMC Pregnancy Childbirth. 2014 Nov 25;14:378. doi: 10.1186/s12884-014-0378-y (PMC4248438; doi:10.1186/s12884-014-0378-y)
Supplement: Additional file 4: — Diet plan page 1. [file 12884_2014_378_MOESM4_ESM.pdf]

DR V SESHIAH
DIABETES RESEARCH INSTITUTE

World Diabetes Foundation

DR V BALAJI
DIABETES CARE CENTRE

DIET CHART / உணவுப்பட்டியல்

Go for walking and do small, simple exercise daily
தினமும் நடைபயிற்சி மற்றும் சிறிய எளிதான உடற்பயிற்சி செய்யவும்

Total Calories /
முழு கலோரி

TIME / நேரம்	MENU / உணவு வகைகள்		Quantity / அளவு
Early Morning<br>காலை<br>6.00-7.00 a.m.	Tea / Coffee without sugar with skimmed milk<br>டீ/காபி சர்க்கரை இல்லாமல் ஆடையில்லாத பாலுடன்		1 கப் (200 ml)
Breakfast<br>காலை டிபன்<br>8.00-9.00 a.m.	இட்லி அல்லது<br>தோசை அல்லது<br>சப்பாத்தி அல்லது<br>பொங்கல் அல்லது<br>உப்புமா அல்லது<br>கோதுமை பிரட் மற்றும்<br>தக்காளி / புதினா சட்டினி<br>அல்லது சாம்பார்	Idly (or)<br>Dosai (or)<br>Chappathi (or)<br>Pongal (or)<br>Uppuma (or)<br>Brown Bread (with)<br>Tomato / Mint Chutney<br>(or) Sambar	3<br>2<br>3<br>1½ கப்<br>1½ கப்<br>3 துண்டுகள்<br>½ கப்<br>1 கப்
Mid-Morning<br>முற்பகல்<br>11.00-11.30 a.m.	ஆடையில்லாத மோர் அல்லது<br>காய்கறி சூப் அல்லது<br>எலுமிச்சை ஜூஸ் மற்றும்<br>மேரி பிஸ்கட் அல்லது<br>அரோரூட் பிஸ்கட் அல்லது<br>காய்கறி சாலட்	Buttermilk (or)<br>Vegetable Soup (or)<br>Lime Juice (with)<br>Marie Biscuit (or)<br>Arrowroot Biscuit (or)<br>Vegetable Salad	1 கப் (200ml)<br>1 கப் (200ml)<br><br>2<br>2<br>1 கப்
Lunch /<br>மதிய உணவு<br>1.00 - 1.30 p.m.	சாதம் அல்லது<br>சப்பாத்தி<br>சாம்பார்<br>ரசம்<br>மோர்<br>கீரை<br>காய்கறி	Rice (or)<br>Chappathi<br>Sambhar<br>Rasam<br>Buttermilk<br>Greens<br>Vegetable	1½ கப் (200ml)<br>2 - 3<br>½ கப்<br>½ கப்<br>1 கப்<br>தாராளமாக<br>தாராளமாக
Evening Snack /<br>மாலை சிற்றுண்டி<br>3.30 - 4.00 p.m.	கோதுமை பிரட் அல்லது<br>சுண்டல் அல்லது<br>அவல் உப்புமா<br>மற்றும் டீ/காபி சர்க்கரை<br>இல்லாமல்<br>ஆடையில்லாத பாலுடன்	Brown Bread (or)<br>Sundal (or)<br>Aval Uppuma (with)<br>Tea / Coffee<br>without Sugar<br>with skimmed milk	2<br>½ கப்<br>½ கப்<br>1 கப் (200ml)
Mid Evening /<br>பிற்பகல்<br>5.30 - 6.30 p.m.	காய்கறி சூப் /<br>ஆடையில்லாத பால்	Vegetable Soup /<br>with skimmed milk	1 கப் (200ml)
Dinner time/<br>படுக்கும் நேரம்	மதிய உணவு போல்	Same as Lunch	
Bed time/<br>படுக்கும் நேரம்	ஆடையில்லாத பால்	with skimmed milk	1 கப் (200ml)
Note / குறிப்பு<br>Weekly once /<br>வாரம் ஒருமுறை	கறி அல்லது Mutton or 2 துண்டுகள் (வாரம் 1 முறை)<br>கோழிக்கறி அல்லது Chicken or 2 துண்டுகள் (75g)<br>மீன் அல்லது Fish or 1 துண்டு (50g)<br>முட்டை Egg or 1 (நாள்தோறும்)		

EXPLORING POSSIBILITIES INDIVIDUALISING NEEDS

729, P.H. Road, Aminjikarai, Chennai-29. Phone # 044-26641414, 26641416 Fax # : 044-2664 0660
e-mail : vseshiah@gmail.com / balajivijayam@gmail.com website : www.drvseshiah.org / www.drbalaji.org
