# Supplementary material for: A cohort study of gestational diabetes mellitus and complimentary qualitative research: background, aims and design
Source: BMC Pregnancy Childbirth. 2014 Nov 25;14:378. doi: 10.1186/s12884-014-0378-y (PMC4248438; doi:10.1186/s12884-014-0378-y)
Supplement: Additional file 5: — Diet plan page 2. [file 12884_2014_378_MOESM5_ESM.pdf]

FOODS TO BE AVOIDED
தவிர்க்கவேண்டிய உணவு வகைகள்

- Conjee (Porridge) as breakfast. கஞ்சி, களி, கூழ், காலை உணவிற்கு தவிர்க்கவும்.
- Roots and Tubers / கிழங்கு வகைகள்
- Sugar, Glucose, Honey, Jaggery, Jam, Cake, Chocolate, ice Cream and Concentrated Milk preparations - Kheer, Koha
  சர்க்கரை, க்ளூகோஸ், தேன், வெல்லம், ஜாம், கேக், சாக்லேட், ஐஸ்கிரீம், திரட்டுப்பால், பாயாசம், பால்கோவா போன்றவை
- Butter, Ghee, Vanaspathi, Coconut Oil, வெண்ணெய், நெய், வனஸ்பதி, தேங்காய் எண்ணெய்
- Nuts - Coconut, Groundnut, Pista, Cashewnuts, Dried Fruits - Raisins
  கொட்டைப்பருப்பு – தேங்காய், வேர்க்கடலை, பிஸ்தா, முந்திரி பருப்பு, உலர்ந்த பழங்கள் – திராட்சை
- Horlicks, Boost, Bournvita, Aerated Drinks ஹார்லிக்ஸ், பூஸ்ட், போன்விட்டா, குளிர்பானங்கள்
- Fatty Mutton, Beef, Pork, Liver, Kidney, Brain, Heart
  கொழுப்பு ஆட்டுக்கறி, மாட்டுக்கறி, பன்றிறைச்சி, ஈரல், சிறுநீரகம், மூளை, இருதயம்.
- Fruits - Banana, Jackfruit, Sapota, Mango, Grapes, Custard Apple.
  பழங்கள் – வாழைப்பழம், பலாப்பழம், சப்போட்டா, மாம்பழம், திராட்சை, சீதாப்பழம்
- Alcohol Especially Beer, Cigarette Smoking. மதுபானங்கள் முக்கியமாக பீர், புகைப்பிடித்தல்.
- Tinned or Canned Foods. பதப்படுத்தப்பட்ட உணவு வகைகள்.

FOODS TO BE INCLUDED
சேர்த்துக்கொள்ளவேண்டிய உணவு வகைகள்

- Greens - கீரைவகைகள்
- Vegetable- Plantain Stem, Plantain Flower, Radish, Cabbage, Bitter Ground, Ladies Finger, Beans, Ridge Gourd, White ash Gourd, Brinjal, Bottle Gourd, Onion, Drumstick, Knol-khol, Cucumber, Tomato, Cauliflower, Pumpkin, Capsicum, Turnip, Chow Chomarrow
  காய்கறிகள் – வாழைத்தண்டு, வாழைப்பூ, முள்ளங்கி, முட்டைகோஸ், பாகற்காய், வெண்டைக்காய், பீர்க்கங்காய், வெள்ளைப்பூசணி, கத்தரிக்காய், புடலங்காய், சுரைக்காய், வெங்காயம், முருங்கைக்காய், நூல்கோல், வெள்ளரிக்காய், தக்காளி, காலி.ஃபிளவர், பரங்கிக்காய், குடைமிளகாய், பீன்ஸ், டர்னிப், சௌசௌ.
- Any one Fruit per day - 1/2 apple, 1 Sweet Lime, 3 Pieces Papaya, 1 Guava when sugar levels are under control.
  ஏதாவது ஒரு பழம் நாள் ஒன்றிற்கு – 1/2 ஆப்பிள், 1 சாத்துக்குடி, 3 துண்டுகள் பப்பாளி 1 கொய்யா – சர்க்கரை அளவு கட்டுப்பாட்டிற்குள் இருந்தால்
- Plain Soda Water / தண்ணீர் சோடா
- Gingelly Oil, Sunflower Oil, Groundnut Oil (Refined) Olive Oil
  நல்லெண்ணெய், சூரியகாந்தி எண்ணெய், கடலெண்ணெய் (ரீ.ஃபைண்ட) ஆலிவ் எண்ணெய்.
- (Use a combination of all these types of oil in an equal ratio for your daily cooking.
  அனைத்து வகை எண்ணெய்களையும் சம அளவில் கலந்து சமையலுக்கு உபயோகிக்கவும்).

World Diabetes Foundation

EXPLORING POSSIBILITIES INDIVIDUALISING NEEDS
