# Supplementary material for: A cohort study of gestational diabetes mellitus and complimentary qualitative research: background, aims and design
Source: BMC Pregnancy Childbirth. 2014 Nov 25;14:378. doi: 10.1186/s12884-014-0378-y (PMC4248438; doi:10.1186/s12884-014-0378-y)
Supplement: Additional file 6: — Depression Assessment Questionnaire. [file 12884_2014_378_MOESM6_ESM.pdf]

Appendix-V

World diabetic foundation and Dr.V.Seshiah Diabetic care & Research institutes Prevention of diabetes in women and children project

Depression assessment questionnaire for GDM post partum mother (Adopted from Edinburgh Postnatal Depression Scale (EPDS)

As you have recently had a baby, we would like to know how you are feeling. Please UNDERLINE the answer that comes closest to how you have felt IN THE PAST 7 DAYS, not just how you feel today.

1. I have been able to laugh and see the funny side of things<br>__As much as I always could<br>__Not quite so much now<br>__Definitely not so much now<br>__Not at all	6. Things have been getting on top of me.<br>__Yes, most of the time I haven't been able to cope at all<br>__Yes, sometimes I haven't been coping as well as usual<br>__No, most of the time I have coped quite well<br>__No, I have been coping as well as ever
**2. I have looked forward with enjoyment to things**<br>**__As much as I ever did**<br>**__Rather less than I used to**<br>**__Definitely less than I used to**<br>**__Hardly at all**	7. I have been so unhappy that I have had difficulty sleeping<br>__Yes, most of the time<br>__Yes, sometimes<br>__Not very often<br>__No, not at all
**3. I have blamed myself unnecessarily with things went wrong**<br>**__Yes, most of the time**<br>**__Yes, some of the time**<br>**__Not very often**<br>**__No, never**	**8. I have felt sad or miserable**<br>**__Yes, most of the time**<br>**__Yes, quite often**<br>**__Not very often**<br>**__No, not at all**
**4. I have been anxious or worried for no good reason**<br>**__No, not at all**<br>**__Hardly ever**<br>**__Yes, sometimes**<br>**__Yes, very often**	9. I have been so unhappy that I have been crying<br>__Yes, most of the time<br>__Yes, quite often<br>__Only occasionally<br>__No, never
**5. I have felt scared or panicky for no very good reason**<br>**__Yes, quite a lot**<br>**__Yes, sometimes**<br>**__No, not much**<br>**__No, not at all**	10. The thought of harming myself has occurred to me<br>__Yes, quite often<br>__Sometimes<br>__Hardly ever<br>__Never

Note: Yellow = EPDS-3 White= EPDS-7 Green =EPDS-2

Score specification:

"no, never" score- 0

Not very often, hardly- 1

Yes quite often- 2

"yes, most of the time" - 3

Overall score: [ ]

Inference:

Score < 10	Score > 10
Not depressed	Depressed<br>Ref to psychiatrist

Data collected on:

Data collected by:

Gestational Age during which data collected: [ ] Months.
