# Supplementary material for: A cohort study of gestational diabetes mellitus and complimentary qualitative research: background, aims and design
Source: BMC Pregnancy Childbirth. 2014 Nov 25;14:378. doi: 10.1186/s12884-014-0378-y (PMC4248438; doi:10.1186/s12884-014-0378-y)
Supplement: Additional file 7: — World Diabetic Foundation and Dr. V. Seshiah Diabetic Care & Research Institutes Prevention of Diabetes in Mother and Children Project - Brief Breastfeeding Questionnaire. [file 12884_2014_378_MOESM7_ESM.pdf]

World Diabetic Foundation and Dr.V.Seshiah Diabetic Care & Research Institutes Prevention of Diabetes in Mother and Children Project - Brief Breastfeeding Questionnaire

1) Are you diagnosed for GDM during pregnancy?

1. Yes (Go to 2)

2. No

2) Have you checked your blood glucose level after delivery and found diabetic

1. Yes
2. No

3) Have you ever breastfed your baby? By breastfeeding, we mean that you have put your baby to your breast, whether or not your baby actually received breast milk, or that you have fed your baby your breast milk.

1. Yes (Go to 4)

2. No (Go to 5)

4) Are you currently feeding your baby your breast milk?

1 .Yes (Go to5)

2. No (Go to 6)

5) Did you breastfeed for as long as you wanted to?

1. Yes

2. No

6) How old was your baby when you stopped breastfeeding?

___ ___# Months, ___ ___ #Weeks, ___ ___ #Days

OR Date stopped: ___ ___/___ __/__ __ __ __ month /day/ year

6. a) which was the most important reason that you stopped breastfeeding?

(Circle one of the following)

1. My baby was hungry and not getting enough milk
2. I had breast pain
3. I wanted to lose weight
4. I was too busy
5. It was too tiring
6. My baby had problems sucking or latching on
7. I was sick or taking medications

8. My baby was not sleeping through the night
9. I planned to return or I returned to work or school
10. My baby was not gaining enough weight with breastfeeding
11. Other_________________________________

7. If you are currently breastfeeding your baby, please answer these questions about: How often you breastfeed your baby during a usual 24 hour time period during the past 7 days. If you are not currently breastfeeding, skip to question 9

7a) In the past 7 days, how many times during the daytime (6AM to 11PM) on average did your baby nurse?

________ Number of times

7b) In the past 7 days, how many times during the night (11PM to 6 AM) on average did your baby nurse?

_________ Number of times

7c) In the past 7 days, how many times did you express (use a pump or manually obtain breast milk) breast milk in an average 24-hour period?

________ number of times ______ number of oz per time (1 ounz= 2tablespoon)

7d) If you expressed breast milk in the past 7 days, how many times did you feed your baby expressed breast milk in an average 24-hour period?

_________ Number of times

8. In the past 7 days, did your baby drink any infant formula?

1. Yes (Go to Question 9)

2. No

9. What type of infant formula/milk do you feed your baby?

1. Infant formula; Brand: _________________________

2. Cow's milk

10. Are your family/friends supportive for breastfeeding?

1. Yes

2. No

11. Do you plan to continue breastfeeding?

If yes, how long? ____________________ If no, why? ______________________________

உலக நீரிழிவு அறக்கட்டளை மற்றும் நீரிழிவு & டாக்டர் பாலாஜி நீரிழிவு பராமரிப்பு ஆராய்ச்சி நிறுவனங்களின் தாய் மற்றும் குழந்தை நீரிழிவு நோய் தடுப்பு ஆராய்ச்சியில் சுருக்கமான தாய்ப்பாலூட்டுதல் சார்ந்த கேள்வித்தாள்

1. நீங்கள் கர்ப்ப காலத்தில் நீரிழிவு நோய்க்கான பரிசோதனை மூலம் நீரிழிவு நோய் இருப்பதாக கண்டறியப்பட்டுள்ளீர்கலா?

1) ஆம் (கேள்வி 2- கு செல்லவும்)

2) இல்லை

2. நீங்கள் பிரசவத்திற்கு பிறகு உங்கள் இரத்த குளுக்கோஸ் அளவை பரிசோதித்து மேலும் நீரிழிவு உள்ளதாக கண்டுபிடிக்கப்பட்டுள்ளீர்கலா?

1) ஆம் என்றால் சர்க்கரையின் அளவு ---------------

2) இல்லை

3. நீங்கள் எப்போதாவது உங்கள் குழந்தைக்கு தாய்பால் கொடுத்துள்ளீர்கலா?தாய்ப்பாலூட்டுதல் என்றால் உங்கள் குழந்தையை மார்பகத்தில் வைத்தல், மற்றும் உங்கள் குழந்தைக்கு தாய்பால் கிடைக்கவில்லை/ கிடைக்கும் இதில் எதுவாயிணும் பரவாயில்லை.

1) ஆம்(கேள்வி 4- கு செல்லவும்)

2) இல்லை(கேள்வி 5- கு செல்லவும்)

4. நீங்கள் தற்போது உங்கள் குழந்தைக்கு உங்கள் மார்பக பாலை கொடுக்கிறீர்கலா?

1) ஆம்(கேள்வி 5- கு செல்லவும்)

2) இல்லை(கேள்வி 6- கு செல்லவும்)

5. நீங்கள் நீண்ட நேரம் போதுமான வரைக்கும் உங்கள் குழந்தைக்கு பால் கொடுக்கிறீர்கலா?

1) ஆம்

2) இல்லை

6. தாய்ப்பால் நிறுத்தப்பட்ட போது உங்கள் குழந்தைக்கு என்ன வயது?

_____மாதங்கள், _____வாரம், _____நாட்கள்
அல்லது தேதி நிறுத்தப்பட்டது: _____ / ____ / _______

(மாதம் / நாள் / ஆண்டு.)

6. a) நீங்கள் தாய்ப்பால் நிறுத்திக்கொண்டதன் மிக முக்கியமான காரணம் என்ன? (ஏதேனும் ஒன்றை வட்டமிடவும்)

1) என் குழந்தை பசியாக உள்ளது மேலும் போதுமான பால் வருவதில்லை

2) எனக்கு மார்பக வலி இருந்தது

3) எனக்கு எடையை இழக்க வேண்டும்

4) நான் பிஸியாக இருந்தேன்

5) அது என்னை மிகவும் சோர்வாக செய்யும்

6) நான் உடல்நிலை சரியில்லாமல் அல்லது மருந்துகள் எடுத்துக்கொண்டு இருந்தது

7) என் குழந்தைக்கு இரவு முழுவதும் தூக்கம் வரவில்லை

8) நான் வேலைக்கு அல்லது பள்ளிக்கு திரும்ப திட்டமிட்டது

9) என் குழந்தைக்கு தாய்ப்பால் போதுமான அளவு எடையை அதிகரிக்கவில்லை

10) இதர காரணம்--

7. நீங்கள் தற்போது உங்கள் குழந்தைக்கு தாய்ப்பால் கொடுக்கிறீர்கல் என்றால் இந்த கேள்விகளுக்கு பதில் சொல்லுங்கள். எவ்வளவு அடிக்கடி நீங்கள் கடந்த 7 நாட்களில், ஒரு வழக்கமான 24 மணி நேர காலத்தில்,

உங்கள் குழந்தைக்கு பால் ஊட்டிணீர்கள்? நீங்கள் தற்போது தாய்ப்பால் கொடுக்கவில்லை எனில், கேள்வி 9 செல்லவும்.

7.a) கடந்த 7 நாட்களில், எத்தனை முறை சராசரியாக (6AM முதல் 11PM வரை) பகல்நேர பொழுதில் உங்கள் குழந்தைக்கு பால் ஊட்டிணீர்கள்?
_______ முறை

7.b) கடந்த 7 நாட்களில், சராசரியாக இரவில் எத்தனை முறை (11PM முதல் 6AM வரை) உங்கள் குழந்தைக்கு பால் ஊட்டிணீர்கள்?
_______ முறை

7.c) கடந்த 7 நாட்களில், எத்தனை முறை நீங்கள் பாலை

வெளிப்படுத்துவதற்கு (மார்பக பால் பெற கைமுறையாக அல்லது ஒரு

பம்ப் பயன்படுத்தி) சராசரியாக 24 மணி நேர காலத்தில்

முயற்சித்தீர்கள்? (1 அவுன்ஸ் = 2 தேக்கரண்டி)

--------------நேரம், ----------- அவுன்ஸ் ஒரு முறை

7.d) கடந்த 7 நாட்களில் மார்பக பாலை வெளிப்படுத்திணீர்கள் என்றால்

நீங்கள் உங்கள் குழந்தை சராசரியாக 24 மணி நேர காலத்தில் எத்தனை

முறை ஊட்டிணீர்கள்?
________ முறை

8) கடந்த 7 நாட்களில், உங்கள் குழந்தை எந்த குழந்தை சூத்திரத்தை குடித்தது?

1)ஆம்(கேள்வி9-குசெல்லவும்)
2) இல்லை

9. உங்கள் குழந்தைக்கு குழந்தை சூத்திரத்தில் / பால் எந்த வகையை ஊட்டிணீர்கள்

9.a) குழந்தை சூத்திரத்தின்; வர்த்தக பெயர்: ____________________________

9.b) மாட்டு பால்

10. உங்கள் குடும்பம் மற்றும் நண்பர்கள் தாய் பாலூட்டலுக்கு ஆதரவாக இருக்கிறார்களா?

1)ஆம்

2)இல்லை

11.உங்கள் குழந்தைக்கு தாய்ப்பாலை தொடர திட்டமிடுவீர்களா? ஆம் எனில், எவ்வளவு காலம்? ______________________ இல்லை என்றால், ஏன்?

____
